# Semi-automated Curation of Metabolic Models via Flux Balance Analysis: A Case Study with *Mycoplasma gallisepticum*

**DOI:** 10.1371/journal.pcbi.1003208

**Published:** 2013-09-05

**Authors:** Eddy J. Bautista, Joseph Zinski, Steven M. Szczepanek, Erik L. Johnson, Edan R. Tulman, Wei-Mei Ching, Steven J. Geary, Ranjan Srivastava

**Affiliations:** 1 Department of Chemical & Biomolecular Engineering, University of Connecticut, Storrs, Connecticut, United States of America; 2 Center of Excellence for Vaccine Research, University of Connecticut, Storrs, Connecticut, United States of America; 3 Department of Allied Health Sciences, University of Connecticut, Storrs, Connecticut, United States of America; 4 Department of Pathobiology and Veterinary Science, University of Connecticut, Storrs, Connecticut, United States of America; 5 Viral and Rickettsial Diseases Department, Infectious Diseases Directorate, Naval Medical Research Center, Silver Springs, Maryland, United States of America; 6 Program in Head & Neck Cancer and Oral Oncology, Neag Comprehensive Cancer Center, University of Connecticut Health Center, Farmington, Connecticut, United States of America; The Pennsylvania State University, United States of America

## Abstract

Primarily used for metabolic engineering and synthetic biology, genome-scale metabolic modeling shows tremendous potential as a tool for fundamental research and curation of metabolism. Through a novel integration of flux balance analysis and genetic algorithms, a strategy to curate metabolic networks and facilitate identification of metabolic pathways that may not be directly inferable solely from genome annotation was developed. Specifically, metabolites involved in unknown reactions can be determined, and potentially erroneous pathways can be identified. The procedure developed allows for new fundamental insight into metabolism, as well as acting as a semi-automated curation methodology for genome-scale metabolic modeling. To validate the methodology, a genome-scale metabolic model for the bacterium *Mycoplasma gallisepticum* was created. Several reactions not predicted by the genome annotation were postulated and validated via the literature. The model predicted an average growth rate of 0.358±0.12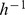, closely matching the experimentally determined growth rate of *M. gallisepticum* of 0.244±0.03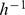. This work presents a powerful algorithm for facilitating the identification and curation of previously known and new metabolic pathways, as well as presenting the first genome-scale reconstruction of *M. gallisepticum*.

## Author Summary

Flux balance analysis (FBA) is a powerful approach for genome-scale metabolic modeling. It provides metabolic engineers with a tool for manipulating, predicting, and optimizing metabolism for biotechnological and biomedical purposes. However, we posit that it can also be used as tool for fundamental research in understanding and curating metabolic networks. Specifically, by using a genetic algorithm integrated with FBA, we developed a curation approach to identify missing reactions, incomplete reactions, and erroneous reactions. Additionally, it was possible to take advantage of the ensemble information from the genetic algorithm to identify the most critical reactions for curation. We tested our strategy using *Mycoplasma gallisepticum* as our model organism. Using the genome annotation as the basis, the preliminary genome-scale metabolic model consisted of 446 metabolites involved in 380 reactions. Carrying out our analysis, we found over 80 incorrect reactions and 16 missing reactions. Based upon the guidance of the algorithm, we were able to curate and resolve all discrepancies. The model predicted an average bacterial growth rate of 0.358±0.12 h^−1^ compared to the experimentally observed 0.244±0.03 h^−1^. Thus, our approach facilitated the curation of a genome-scale metabolic network and generated a high quality metabolic model.

## Introduction

The ability to determine a relationship between an organism's genome and its molecular physiology quantitatively, based upon current understanding of metabolism and molecular biology, continues to improve at a dramatic pace. Establishment of a quantitative model allows for the compact and integrated analysis of complex metabolic networks and their associated and often non-intuitive behavior. Genome-scale metabolic modeling in particular has been used in a number of venues in the biotechnological and biomedical arenas [1]–[19].

Genome-scale metabolic modeling facilitates a deep understanding of metabolism of microorganisms. Modeling is also a tool to elucidate missed information and recognize errors in new and existing models of metabolic networks. As was pointed out by Thiele *et al.* [20], the quality of a modeled metabolic network depends on the available information and the procedure used for the reconstruction. Additionally, the reconstruction process has several challenges associated with it. For example, the use of incomplete or inaccurate genome annotation will result in an incomplete model description. The presence of some reactions in the model may be missing due to lack of knowledge regarding the function of specific proteins [21]. Furthermore, the interactions among enzymes and the reactions they catalyze may be quite complex. For instance, one reaction may be catalyzed by many different enzymes (isoenzymes). Conversely, one enzyme may catalyze several different reactions [22], [23]. Moreover, adequate information regarding the localization of metabolites within the cell may not be known. In other cases, the directionality of a reaction may need to be elucidated [24]. Finally, the information regarding the biomass composition and the energy requirements of the organism may not be known. As a result, these data have to be determined from experiments and/or they must be estimated from what is known about closely related species [25]–[27]. Despite this effort, it still may not be possible to generate results comparable with experimental findings due to undiscovered errors or missing information.

Metabolic network modeling is an iterative process that starts with the generation of a genome-scale reconstruction of metabolism utilizing all current annotations and literature information. Next, curation of the draft reconstruction is carried out manually by an expert, and the computational model is generated. Finally, a comparison of the *in silico* results relative to the experimental information is assessed. The iterative process stops when the results from the model are nearly the same as the experimental results. In the future, as more information becomes available, the model may be updated in order to fill all the possible gaps and achieve better results [20], [22], [26]. Most genome annotations are carried out using automated techniques, many of which rely on pairwise similarity scoring of predicted proteins to those in databases [26], [28]–[30]. Such a process is imperfect, as the ability to reliably annotate a given protein with a specific function can be greatly impacted by sequence divergence, lack of reliable functional knowledge, or annotation for sequence matches. This currently results in genome annotations that may not accurately or completely reflect the organism's metabolic functions. The use of metabolic network modeling provides an additional and powerful methodology to help compensate for these gaps in knowledge, leading to a more accurate reconstruction of an organism's metabolism.

The model curation process improves the draft reconstruction by identifying and filling the gaps present in the network, removing reactions that are not likely present in the organism, and enforcing overall consistency across the network. Some computational strategies for identifying missing reactions and filling the gaps have been developed previously, such as GapFind [29], GapFill [29], the Pathway Tools Hole Filler [31], the “metabolic expression placement” [32], [33], MetaFlux [34], Model SEED [35]. GapFind is an optimization-based procedure to identify missing reactions in the network. Gaps can be filled with GapFill by adjusting the existing metabolic network through reversing the directionality of existing reactions; adding transport reactions between compartments; adding exchange fluxes; or by adding a minimum number of reactions from a reference database [36]. MetaFlux is part of the Pathway Tools software for generating FBA models. It uses a multiple gap filling approach based on a mixed integer linear programing (MILP) to suggest reactions to be added from the MetaCyc database, identify biomass metabolites which are required but can not be produced, and choose nutrient and secretion fluxes to be added to the model from a “try set” defined by the user. Model SEED is a web-based resource for the creation of new metabolic models. After a preliminary reconstruction model is created in Model SEED, an auto-completion step is performed by using an MILP algorithm that identifies the minimal set of reactions from the SEED reaction database that must be added to fill the gaps present in the network. However, all these approaches are dependent upon an existing reference database of information to resolve these curation issues.

Here a new algorithm for facilitating curation of models is presented. The approach integrates a genetic algorithm (GA) with flux balance analysis. The novelty and strength of this GA/Flux Balance Analysis (GAFBA) strategy lies in its ability to both aid in fundamental studies of metabolism and to facilitate curation of genome-scale metabolic networks, rather than functioning solely as a predictive tool. Furthermore, the strategy is independent of a reference database, allowing the researcher to investigate other avenues of curation. However, this approach does not preclude the use of existing databases as one of those sources of information. Rather it provides increased flexibility in evaluating the system of interest. As the quality of curation increases, the evolved model can be used as a predictive tool, but that is a secondary contribution of this approach.

The premise of the GAFBA method is based upon the observation that the optimization of an initial genome-scale metabolic model often results in no feasible solution when experimental information is incorporated. This result usually indicates that some of the constraints cannot be satisfied. The GAFBA method identifies the mass balanced constraints that may be relaxed to solve the FBA optimization problem. If relaxing a selected constraint allows the problem to be solved, it is likely that the associated metabolite was participating in a metabolic reaction that, due to lack of information during the reconstruction process, was not taken into account in the model. As a result, the metabolite and the potential reactions it may participate in should be reviewed. The approach developed here employs a genetic algorithm to identify the minimum number of constraints that could be relaxed in order to achieve a feasible solution to the FBA optimization problem.

To develop and evaluate this strategy, *Mycoplasma gallisepticum* was chosen as the model organism. The choice of *M. gallisepticum* for the validation of the GAFBA methodology was driven by our expertise with the organism and the organism's importance to the poultry industry. *M. gallisepticum* causes chronic respiratory disease in chickens and infectious sinusitis in turkeys [37]. The disease can be easily propagated by direct or indirect means. *M. gallisepticum* has been a concern to the poultry industry because of the resultant increase in chick and poult mortality, reduced egg production, and increased costs related to medication, prevention and control programs. For example, in 1994, the layer industry alone lost between $118 and $150 million in the United States [38]. A better understanding of *M. gallisepticum* biology, including virulence, genomics, and metabolic processes has the potential to allow for the development and improvement of the vaccines and control strategies for this disease.

In this work, the metabolic network of *M. gallisepticum* was developed by using the organism's annotated genome sequence, compiling existing enzymatic data, employing genome-scale bioinformatics-driven homology searches, referencing the metabolism of other closely related *Mycoplasma* strains, generating a comprehensive biomass equation, and finally analyzing how all the metabolites in the system interacted with each using GAFBA algorithm.

## Results

### Hybrid Genetic Algorithm/Flux Balance Analysis (GAFBA)

**Figure 1:**
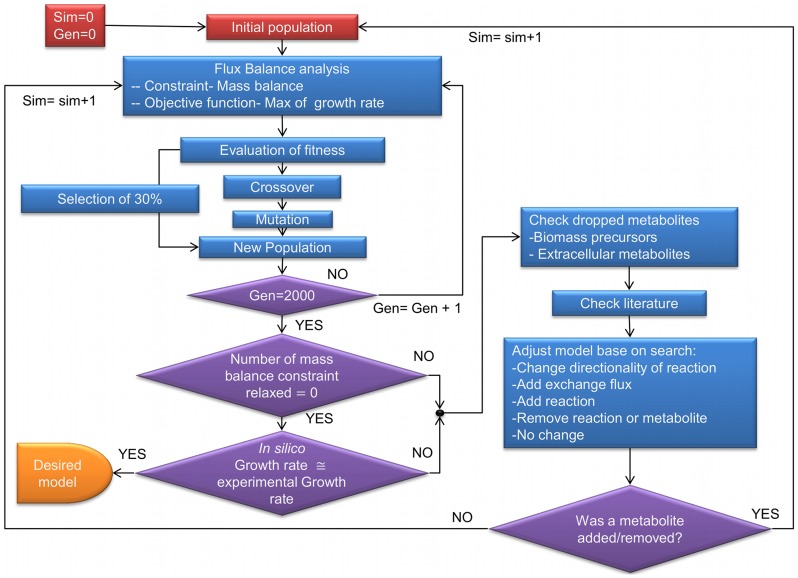
Flowchart for GAFBA algorithm. A schematic depiction of the GAFBA algorithm used to determine the genome-scale metabolic model for *M. galliscepticum*.

The hybrid Genetic Algorithm/Flux Balance Analysis Algorithm (GAFBA) algorithm embeds the Flux Balance Analysis (FBA) optimization problem within a genetic algorithm to identify problematic metabolic constraints and is schematically depicted in Figure 1. The optimization problem was resolved through a hierarchical approach, with the minimization of the number of relaxed constraints being given primary importance. The pool of models that had the same number of constraints relaxed were then further discriminated against by determining which individual models had the highest growth rate values, a frequently used objective function for FBA [39]–[42]. After each simulation, a list of problematic metabolites, where “problematic metabolites” refer to metabolites on which mass balance constraints were forced to be relaxed, was generated to help to elucidate potential errors or missing information. Based on these data, it was possible to decide the best manner by which to fill in the missing information if possible. A revised version of the mechanisms to solve gaps previously presented by Maranas' group was applied [29]. The options were: 1) change the directionality of a reaction, 2) add an exchange flux for the metabolite, 3) add a transport or intracellular reaction, 4) remove a reaction or metabolite from the model, or 5) no change. Examples for each case are presented in the supplementary file Text S1. The option chosen was based on experimental data, literature data or information from related organisms.

The initial genome-scale metabolic model was based upon an updated annotation of the *M. gallisepticum* genome [43]. Using the Pathway tools software platform [44], a Pathway Genome Database was constructed that accounted for all metabolic pathways as determined from the genome annotation.

The GA started with the random generation of an initial population of data structures referred to as “chromosomes” per the GA parlance [45], [46]. Each chromosome represented a potential metabolic model of the microorganism. The chromosomes were binary encoded where each of the genes represented a mass balance constraint. If the mass balance constraint for a metabolite was relaxed, the gene was assigned a value of zero. If it was enforced, the gene was assigned a value of one. The number of mass balance constraints that were relaxed in the initial population was determined randomly. The population was then allowed to evolve using the genetic operations of reproduction, crossover, and mutation in order to achieve a fitter population and ultimately an accurate metabolic model. The objective function for GAFBA was to minimize the number of mass balance constraints relaxed. However, it was subject to the constraint that the FBA model return a feasible solution.

A list of the problematic metabolites was generated upon completion of the run. Problematic metabolites were defined as those that were unable to fulfill the mass balance under the given conditions; manual review of each of the metabolites, as well as some of the upstream and/or downstream metabolites in the associated pathway was required. The analysis of the metabolites upstream and/or downstream in the pathway helped to determine if the problem could be corrected by adjusting the mass balance of a closely connected metabolite instead of the problematic metabolite directly. For example, it is possible that one of the downstream reactions responsible for consuming the problematic metabolite is blocked because the reaction is producing a dead end metabolite. Thus, it is necessary to adjust the mass balance of the dead end metabolite and not the mass balance of the so-called problematic metabolite. An example of an upstream issue is when a reaction responsible for producing the problematic metabolite has no flux due to the lack of a reactant. Under such conditions, the mass balance for the reactant has to be adjusted rather than the mass balance of the initially identified “problematic” metabolite.

After updating the model, a new simulation was started. If a metabolite was added or removed, a new initial population was generated randomly and evolution was allowed to proceed. If no metabolite was added or removed, GAFBA could either be continued from where it left off using the final population from the previous run as the seed population, or an entirely new population could be generated. The evolutionary process was then re-started. The ultimate termination criterion was when none of the mass balances constraints needed to be relaxed.

### Initial reconstruction of the *M. gallisepticum* metabolic model

The first step in the reconstruction of a genome-scale metabolic model of *M. gallisepticum* was to analyze the published annotation of Strain R Clone 2 [43] using the Pathway Tools Pathologic automated metabolic network generation platform [47]. Pathway Tools Pathologic compiled reactions associated with Enzyme Commission (EC) numbers and enzymes listed in the annotation [44]. Next, an in-depth literature review focusing on previously elucidated enzymatic activity was performed. Reactions shown to be active in *M. gallisepticum* were subsequently added. Reactions shown to be inactive were removed. Once the pool of published enzymatic data had been exhausted, the Pathway Tools Hole Filler was used to generate a list of gene candidates to potentially fill gaps in various metabolic pathways.

Any candidate reaction from Pathway Hole Filler with a probability assignment over 0.9 was selected for further evaluation. Selected candidates were evaluated by a curator based on a number of criteria. First, presence or absence of a candidate reaction's associated pathway in evolutionarily related species such as *Mycoplasma genitalium*, *Mycoplasma pneumoniae*, and other members of the *Mycoplasma* family was taken into account. Moreover, it considered was whether or not the products of a given pathway were known to be present in the cell biomass, utilized by another pathway, or secreted from the cell. Reactions whose products were not utilized or secreted were considered unlikely to be present. Additionally, candidate reactions experimentally proven to be absent in *Mycoplasma gallisepticum* metabolism were removed.

A total of 23 reactions were added due to supporting experimental data and are listed in Table 1. Five total reactions, shown in Table 2, were added based on BLASTP homology results, and 12 high confidence reactions were added due to results of the Pathway Hole Filler analysis and are shown in Table 3. Two reactions catalyzed by annotated enzymes were found in previous studies not to be present in *M. gallisepticum*. These reactions were subsequently removed and are listed in Table 4. Taking these modifications into account, the initial model consisted of 446 metabolites involved in 380 reactions.

**Table 1:** Summary of reactions added to the metabolic model based on experimental data in the literature.

Enzyme Name	EC #	Associated Gene	Citation
HMG-CoA Synthase	2.3.3.10	?	[105]
CoA transphorase	2.8.3.8	?	[105]
Membrane DNAases and RNaases	None	?	[106]
Succinyl CoA: Acetoacetate CoA-transferase	2.8.3.5	?	[105]
HMG-CoA Reductase	1.1.1.34	?	[105]
Malate synthase	2.3.3.9	?	[107]
Pyruvate Carboxylase	6.4.1.1	?	[107]
AMP phosphatase	3.1.3.5	?	[108], [109]
GMP phosphatase	3.1.3.5	?	[108]–[110]
dAMP	3.1.3.5	?	[111]
Adenylosuccinate synthetase	6.3.4.4	?	[112]
Adenylosuccinate lyase	4.3.2.2	?	[112]
Deoxyadenosine kinase (ATP-dependent)	2.7.1.76	MGA_0174, MGA_0175	[110]
Deoxyguanosine kinase (ATP-dependent)	2.7.1.133	MGA_0174, MGA_0175	[110]
Deoxycytidine deaminase	3.5.4.14	MGA_0361	[31], [113]
Uridine phosphorylase	2.4.2.3	?	[31], [109], [113]
Deoxyuridine phosphorylase	2.4.2.23	?	[109]
Uracil phosphorylase	None	MGA_0362	[31], [109]
Malate dehydrogenase	1.1.1.37	MGA_0746	[107], [114], [115]
Ribose-5-phosphate isomerase	5.3.1.6	MGA_0886	[110], [116]
Asparate aminotransferase	2.6.1.1	?	[117]
Serine hydroxymethyltransferase	None	MGA_1146	[118]
Phospholipase A1	3.1.1.32	?	[100]

Enzyme names normally catalyzing reactions described in the literature and corresponding E.C. assignments are listed. All EC numbers were determined via Pathway Tools v14. *M. gallisepticum* genes potentially associated with these activities are noted, and enzymes/activities lacking gene associations of confidence are indicated with question marks.

**Table 2:** Summary of reactions added based on BLASTP analysis.

Enzyme name	EC #	M. gal Locus	Organism	Locus	Forward E value	Reverse E Value	Citation
1,2 diacylglycerol 3-B-galactosyltransferase	2.4.1.46	MGA_0001	*M. pneumonaie*	mpn483	3.E-15	7E-13	[119], [120]
Galactolipid galactosyltransferase	2.4.1.184	MGA_0001	*M. pneumonaie*	mpn483	3.E-15	7.E-13	[119], [120]
Phosphoglycerate kinase (dGTP)	2.7.2.3	MGA_1187	*M. pneumonaie*	mpn429	1.E-128	1.E-128	[51], [121]
Phosphoglycerate kinase (GTP)	2.7.2.10	MGA_1187	*M. pneumonaie*	mpn429	1.E-128	1.E-128	[51], [121]
Phosphopentomutase	5.4.2.7	MGA_0358	*M. pneumonaie*	mpn066	6.E-120	7.E-120	[51]

Listed here are *M. gallisepticum* genes found by forward and reverse BLASTP [58], [59] searches to be significantly similar to genes in related mycoplasmas that catalyze the corresponding listed reactions. These reactions were added to the model. All EC numbers were determined via Pathway Tools v14.

**Table 3:** Reactions added based on Pathway Tools analysis.

Gene	Annotated Gene Function	New HF Gene Function	Hole EC#	Ptools HF Probability	Additional Citation/Rationale
MGA_0008	Putative Glycerol-3-phosphate acyltransferase	Glycerol-3-phosphate O-acyltransferase	2.3.1.15	0.98	Needed for glycerol incorporation for phospholipid biosynthesis
MGA_0161	Dihydrolipoamide dehydrogenase (E3) component of PDH complex	Glycine Decarboxylase	None	1.00	Folate interconversion
MGA_0161	Dihydrolipoamide dehydrogenase (E3) component of PDH complex	NAD(P)(+) Transhydrogenase (B-specific)	1.6.1.1	0.99	Needed for NADP charging
MGA_0181	Fatty acid/phospholipid synthesis protein Plsx	Acyl-Phosphate Synthase	None	0.99	Needed to provide an acyl carrier protein for lipid metabolism
MGA_0291	Inorganic polyphosphate/ATP-NAD kinase	NADH Kinase	2.7.1.86	0.96	Needed for NADH metabolism
MGA_0364	Purine nucleoside phosphorylase deoD-type	Deoxyinosine phosphatase	None	0.99	[109]
MGA_0594	Glutamyl-tRNA synthetase (Glutamate—tRNA ligase) (GluRS)	Glutamine tRNA ligase	6.1.1.18	1.00	Necessary tRNA charging pathway
MGA_0594	Glutamyl-tRNA synthetase (Glutamate—tRNA ligase) (GluRS)	Glutamine tRNA ligase	None	0.92	Necessary to the glutaminyl-tRNA charging pathway
MGA_0596	Bifunctional protein folD	Methylenetetrahydrofolate dehydrogenase (NAD+)	1.5.1.15	1.00	Homology to *Escherichia coli's* FolD which catalyzes this reaction
MGA_0833	Acetyl-CoA hydrolase	Acetate CoA transferase	2.8.3.8	0.98	[105]
MGA_0950	Guanosine polyphosphate pyrophosphohydrolases/synthetase	GTP-pyrophosphokinase	2.7.6.5	1.00	Needed for ppGpp Biosynthesis
MGA_1065	Asparaginyl-tRNA synthetase	Asparate tRNA ligase	6.1.1.-	1.00	Needed for L-asparginyl tRNA charging pathway

This table shows the genes, previously annotated functions, newly annotated functions, reaction EC numbers, and HF probability and the rationale for why they were added. All EC numbers were determined via Pathway Tools v14. It should be noted that the functionalities listed here are in addition to the original functionality of the given gene.

**Table 4:** Reactions removed based on experimental studies.

Removed due to experimental evidence			
Enzyme name	EC #	Associated gene	Citation
Deoxyribose-5-phosphate aldolase	4.1.2.4	MGA_0363	[116]
dUTPase	3.6.1.23	MGA_0994	[111], [122]

Here the reactions and associated enzymes that were shown to be absent in *M. gallisepticum* based on previous experimental studies and therefore not incorporated into the model are listed.

### Refinement of the initial reconstruction

FBA of the initial *M. gallisepticum* model resulted in no feasible solution. Thus, the model was analyzed using GAFBA to identify reaction holes, unproduced but necessary biomass components, and metabolites with no membrane transporter or degradation pathway. The biomass equation was meticulously investigated. A large number of citations were accumulated for many metabolites that were known to be biosynthesized by *M. gallispeticum* but were not accounted for based on the genome annotation. For example, phosphatidylcholine, cardiolipin, and sphingomylin are all phospholipids known to have working biosynthesis pathways in *M. gallispeticum* because of previously conducted fatty acid radioactive labeling assays [48]. Nevertheless, there are a number of reactions necessary to biosynthesize these components that are absent in *M. gallispeticum*. Therefore, logical reactions were assigned to complete these biosynthesis pathways, usually by referencing similar pathways in other *Mycoplasma* genomes. However, it is critical to note that the rationale for including these pathways was to complete the model. It does not constitute proof of existence of that pathway. That being said, the requirement of such pathways for the model does open up interesting experimental questions and new hypotheses.

The GAFBA algorithm also revealed a number of metabolites that were not degraded and could not be transported out of the cell. A total of 16 reactions were added based on the results of the first GAFBA run. A list of all of these added reactions, along with a brief description of the rationale for the addition and all relevant citations are shown in Table 5.

**Table 5:** Initial model modifications.

Enzyme Name	EC #	Needed Product/Un-degraded Metabolite	Rationale	Citation
Pyruvate kinase	2.7.4.6	DNA	Needed for DNA synthesis. 7 reactions total of this EC# added.	[121]
Phosphatidate phosphatase	3.1.3.4	A phosphotidyl-choline	Needed to synthesize a lipid experimentally proven to be biosynthesized	[48]
Phosphatidylglycerophosphatase	3.1.3.27	Cardiolipin	Needed to synthesize a lipid experimentally proven to be biosynthesized	[48]
Unnamed	2.7.8.-	Cardiolipin	Needed to synthesize a lipid experimentally proven to be biosynthesized	[48]
Chlorinephosphate cytidylytransferase	2.7.7.15	A phosphotidyl-choline	Needed to synthesize a lipid experimentally proven to be biosynthesized	[48]
Diacylglycerol chlorinephosphotransferase	2.7.8.2	A phophotidyl-choline	Needed to synthesize a lipid experimentally proven to be biosynthesized	[48]
Sphingomyelin Synthase	2.7.8.27	A sphingomyelin	Needed to synthesize a lipid experimentally proven to be biosynthesized	[48], [100]
PNPase	3.1.3.7	Adenosine 3′,5′-biphosphate	Biphosphate, the byproduct of the acyl carrier protein charging reaction necessary for fatty acid utilization	[48], [123], [124]
Fatty acid acyl group creator	6.2.1.20	Acyl-fatty acid	Needed for fatty acid assimilation	[48], [123], [124]
Maltose phosphorylase	2.4.1.8	B-D-glucose-6-phosphate	Needed for maltose degradation	[125]
Serine hydromethyltranferase	None	5-methyl-tetrahydrofolate	Tetrahydrofolate	[118]
Pyridoxamine kinase	2.7.1.35	Pyridozyl 5′-phosphate	Needed for vitamin B6 production	[51]
Fructose-1-phosphate kinase	2.7.1.89	Fructose-1,6-biphosphate	Fructose degradation essential	[125]
Thiamine kinase	2.7.1.89	Thiamine diphosphate	From thiamine	[51]
Thiamine-monophosphate kinase	2.7.4.16	Thiamine diphosphate	Needed to complete vitamin b1 biosynthesis from thiamine	[51]
Adenosylhomocysteinase	3.3.1.1	S-adenosyl-L-homocysteine	Needed to degrade the S-adenosyl-L-homocysteine formed from tRNA methylation	none

The first set of changes made to the preliminary model based on GAFBA results are provided. Rationale and relevant citations are listed for each.

Despite application of the changes shown in Table 5, the modeled and experimental mass balances remained inconsistent. Using GAFBA, additional changes were needed in order to fulfill the mass balance and additional runs continued to require refinement of the model. The remaining modifications are presented in the Table 6 with the details described below.

**Table 6:** Remaining changes made to model.

Metabolite	compartment	change	reference
Charged tRNA's	cytosol	added recycling rxn to biomass	[51]
Uncharged tRNA's	cytosol	added recycling rxn to biomass	[51]
Oxygen	cytosol	added exchange flux	Experimental conditions
Cytidine	cytosol	added exchange flux	[50]
Hydrogen peroxide	cytosol	added exchange flux	
Carbon dioxide	cytosol	added exchange flux	
Chloride	cytosol	added exchange flux	[50]
L-alpha-alanine	cytosol	added exchange flux	[50]
L-cysteine	cytosol	added exchange flux	[50]
L-threonine	Cytosol	added exchange flux	[50]
L-glutamine	Cytosol	added exchange flux	[126]
L-aspartate	Cytosol	added exchange flux	[50]
Glycine	Cytosol	added exchange flux	[50]
all the rest of amino acids	Extracellular	added exchange flux	[50]
Ceramides	Extracellular	added exchange flux	
Biomass	Cytosol	GAM was calculated and added to biomass equation	[20]
Ribose-5-phosphate	Cytosol	changed the directonality of E.C. 5.3.1.6	KEGG [60]
Ribose-5-phosphate	Cytosol	changed the directonality of E.C. 5.1.3.1	KEGG [60]
Thymidine	Cytosol	changed the directonality of E.C. 2.4.2.4	KEGG [60]
Sodium ion	Extracellular	added exchange flux	[50], [127]
Formate	Cytosol	added E.C. 1.2.1.2 rxn	[51]
a protein L-methionine	cytosol	remov general rxn RXN-8668	
Choline	Cytosol	added E.C. 3.6.3.7	[57]
dCTP	Cytosol	Changed the direction of E.C. 1.8.1.9	KEGG [60]
NAD+	Cytosol	Added E.C. 6.3.5.1	[49], [50]

The cumulative changes from the second and succeeding rounds of analysis done with the GAFBA algorithm are presented.

Through use of GAFBA, it was found that the mass balance on the charged tRNA's and uncharged tRNA's could not be enforced under the given metabolic description. The discrepancy was due to uncharged tRNA's being converted to charged tRNA's by the tRNA's ligase reactions but participating in no other reactions. This ultimately resulted in the depletion of uncharged tRNAs if the mass balance was enforced without modification. To rectify this imbalance, another reaction was added representing a recycling process to convert the charged tRNA's to uncharged tRNA's and complete the cycle. These reactions were added to the biomass equation. This was similar to the approach taken for *M. genitalium* model *i*PS189 [49], which also includes charged and uncharged tRNA molecules in the biomass equation.

Because the growth medium used experimentally was undefined, many of the necessary exchange fluxes for the model could not be explicitly resolved. The decision to add an exchange flux to the model was based on experimental information and on the composition of defined media for relative species, such as *M. genitalium* [49] and *M. laidlawii* [50]. All added exchange fluxes are listed in Table 6.

Sufficient experimental data was unavailable to directly calculate the value for the growth associated maintenance (GAM) and non-growth associated maintenance (NGAM) for *M. gallisepticum*. Thus, the GAM of *M. pneumonia* [51] was added to the biomass equation, and the value for NGAM was taken from the *M. genitalium i*PS189 model [49].

The reaction catalyzed by the enzyme formate dehydrogenase (E.C. 1.2.1.2) was added to the model in order to complete the mass balance on formate. This reaction is present in other *Mycoplasmas* [51], and based on the GAFBA results. It was hypothesized that this reaction was also part of *M. gallisepticum* metabolic network.

**Figure 2:**
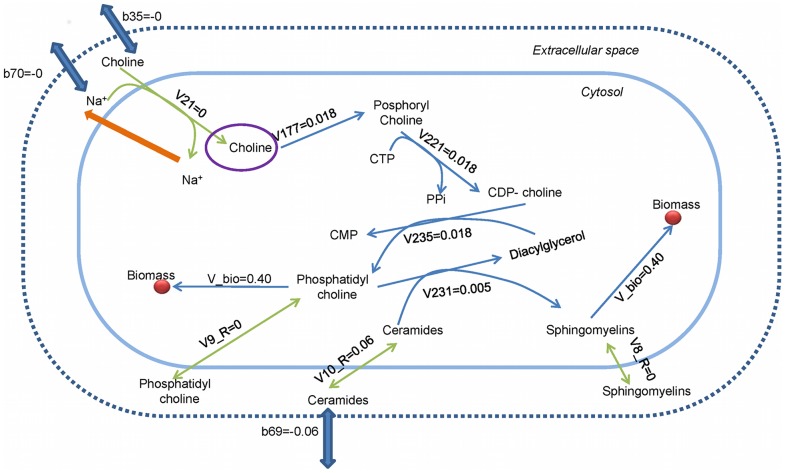
Choline subnetwork. The purple circle shows the unconstrained metabolite. The thin blue arrows are the standard fluxes, while the thick blue arrows are the exchange fluxes. The orange arrow represents the proposed solution. The solid blue line is the plasma membrane and the dashed blue line is the system boundary. The red circle is an abstraction of the biomass pool.

Another problematic metabolite identified by GAFBA that was hindering the successful simulation of the metabolic network was choline. The *M. gallisepticum* model required choline for the production of phosphatidyl choline, which was ultimately necessary for biomass. However, although the reaction for consumption of choline was present, no choline was predicted to enter the cell. The transport reaction for choline, represented by flux V21 in Figure 2, was coupled with sodium ion transport based on the reconstruction. When choline was transported from the extracellular space to the cytosol, it co-transported a sodium ion. The issue was that the sodium ion in the cytosol was not used in any other reaction. To avoid accumulation of unused sodium in the cell, the model predicted that transport reaction (V21) would be zero. A possible solution for this issue was to add a reaction that used sodium ions. In reviewing the literature, it was found that sodium is used for volume regulation in *Mycoplasma* [52]–[57] via translocation from the cytosol to the extracellular environment by a Na-ATPase (E.C. 3.6.3.7). Searching the *M. gallisepticum* proteome for Na-ATPase using BLASTP [58], [59] and the *Aspergillus fumigatus* Af293 sodium P-type ATPase (GenBank Accession no. XP_751881.1) as probe, the best match (82% percentage coverage, score of 306, and E-producing value of 5.E-91) was identified at locus NP_853020.2, the previously annotated cation transported ATPase MGA_1061.

The mass balance for dCTP was also required to be unconstrained based on GAFBA. It was initially predicted that more dCTP was produced from dCDP than was consumed by the biomass reaction. dCDP was produced from CDP coupled with reduced thioredoxin and oxidized NrdH glutaredoxin-like protein. When the metabolic networks of these metabolites were checked, the thioredoxin reductase reaction (E.C. 1.8.1.9) was found to be reversed [60]. Correcting E.C. 1.8.1.9 directionality resolved the dCTP problem.

The last two metabolites for which the mass balance constraints were relaxed were 2-phospho-4-{cytidine 5′-diphospho}-2-C-methyl-D-erythritol and nicotinamide adenine dinucleotide (NAD+).

2-phospho-4-{cytidine 5′-diphospho}-2-C-methyl-D-erythritol is a metabolite in the methylerythitol phosphate pathway (MEP). It is used for the production of isopentenyl diphosphate (IPP) and dimethylallyl diphosphate (DMAPP), the two precursors of isoprenoid. For many years, it was assumed that all organisms produced IPP from acetyl-CoA through the mevalonic acid pathway (MVA), and then IPP was isomerized to DMAPP [61]–[63]. However, an alternative pathway was reported for the production of the building blocks of isoprenoid in bacteria and plants [64]–[67]. Although studied in a variety of mycoplasmas, results regarding the presence or even partial presence of the MEP pathway have yet to be resolved. Some labs have reported not finding any of the genes encoding for the MEP pathway [68]–[70], while other labs found that mycoplasmas have portions of the MEP pathway [63], including *M. penetrans* [71] and *M. gallisepticum* [72]. The MEP pathway in the *M. gallisepticum* model ended in the production of (E)-4-hydroxy-3-methyl-but-2-enyl pyrophosphate (HMB-PP), a dead-end metabolite in the model. No gene was associated to the reaction HMB-PP reductase (E.C. 1.17.1.2) responsible for converting HMB-PP to IPP. Two questions that remain are whether *M. gallisepticum* has a complete MEP pathway and what is the purpose of the HMB-PP metabolite.

The second constraint that required relaxing was that of the NAD+ mass balance. NAD+ is recovered by passing the electrons from NADH to oxygen and lactate but these mechanisms are insufficient to recover NAD+ in the model. Thus a reaction to synthesize NAD+ was required. *M. gallispeticum* had a partial NAD+ salvage pathway. The partial pathway starts with the conversion of nicotinate to nicotinate mononucleotide catalyzed by the enzyme nicotinate phosphoribosyltransferase (E.C. 2.4.2.11). Then nicotinate mononucleotide is converted to nicotinate adenine dinucleotide through the reaction catalyzed by nicotinate-nucleotide adenyltransferase (E.C. 2.7.7.18). Next nicotinate adenine dinucleotide is converted to NAD+ by NAD+ synthethase (E.C. 6.3.5.1), but no gene was found for this enzyme in *M. gallisepticum*. Interestingly, *M. pneumonia* uses reaction NAD+ synthetase to synthesize NAD+, but blasting this gene against *M. gallisepticum* shows no close homology. One more option was that NAD+ was taken from the media, but no literature evidence was found about the requirements of NAD+ [73]. Instead, the media composition of *M. genitalium* [49] and *M. laidlawii* [50] had nicotinate, the starting component of the partial NAD salvage pathway. Based on the observation that *M. gallisepticum* had two reactions of the partial pathway and the requirement of the starting component in other relatives, it was hypothesized that NAD+ synthetase reaction was present in *M. gallisepticum*. However, more experimental and computational work is clearly required to validate this theory.

Interestingly, when the NAD+ synthetase reaction was added to the model, it was possible to enforce all the mass balance constraints in the model. The mass balance of 2-phospho-4-{cytidine 5′-diphospho}-2-C-methyl-D-erythritol was consistent after adding NAD+ synthethase because the MEP pathway no longer had any flux going through it.

### Final model

**Figure 3:**
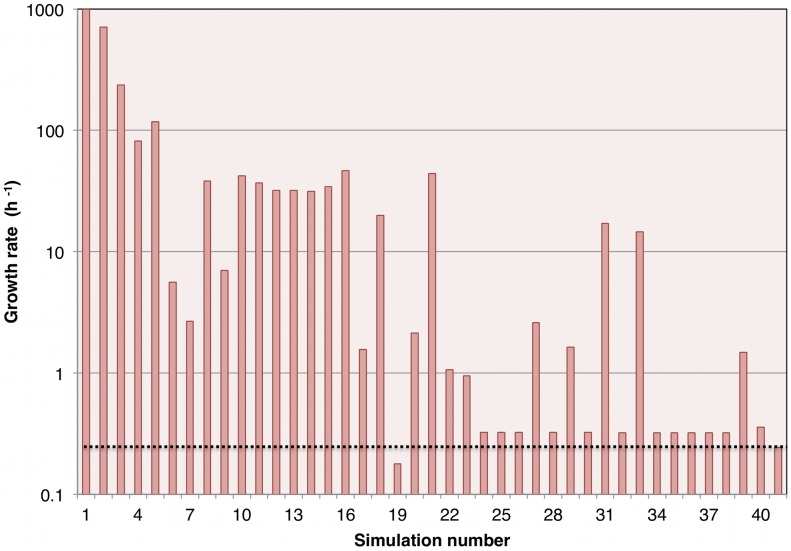
Predicted growth rate over the course of model evolution. 40 simulations were carried out for 2,000 generations each. The columns represent the growth rate values. Result 41 is the average value of the experimentally measured *M. gallisepticum* growth rate. The dashed line running the length of the graph also indicates the average experimentally measured growth rate and is shown as a reference to facilitate comparison with the simulation results.

**Figure 4:**
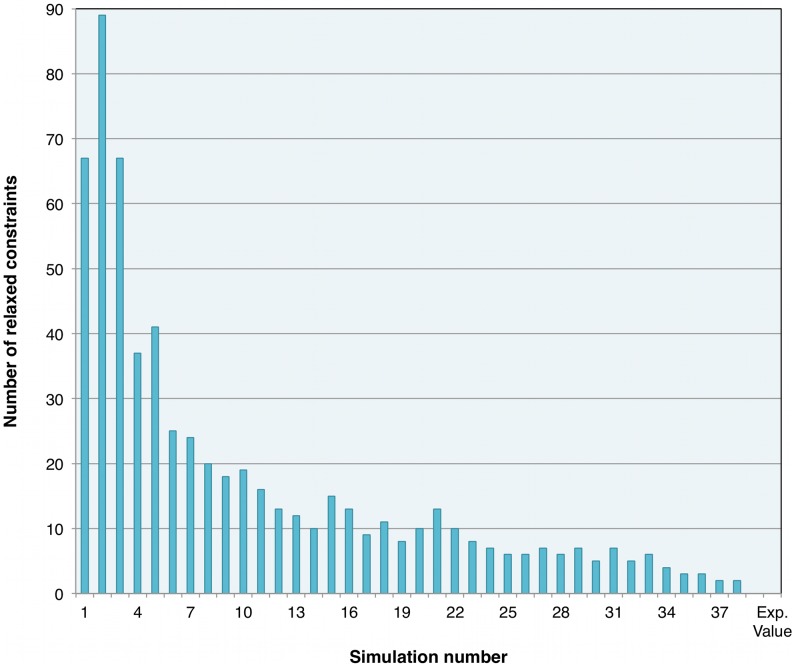
Metabolic constraints relaxed over the course of model evolution. 40 simulations were carried out for 2,000 generations each. The columns represent the number of mass balances for which the constraints were relaxed.

The final model was obtained after 40 runs of GAFBA with each run consisting of 2,000 generations. Figure 3 shows the results for each of the simulations and the corresponding values of growth rate, and Figure 4 shows the number of mass balance constraints that were relaxed. The best model, represented by simulation 40, was able to close all the mass balances constraints and had a growth rate value of 0.358±0.12 h^−1^ compared with the experimental value of 0.244±0.03 h^−1^. The final *M. gallisepticum* model accounted for 441 metabolites and 395 reactions. The model had 234 intracellular reactions; 86 transport reactions for the transfer of metabolites between the extracellular space and the cytosolic compartment; and 73 exchange reactions that allowed the uptake or secretion of metabolites either to or from the system boundary. A complete description of the model is provided in an Excel format in supplementary file Data S1 and in SBML format in the supplementary file Data S2. A copy of the source code for the model is provided in the supplementary file Data S3. The model may be run using the open source GNU Linear Programming Kit (http://www.gnu.org/software/glpk/).

### Comparison of *M. gallisepticum* model and *M. genitalium i*PS189 model

To provide a better context for *M.gallisepticum* model, it is compared to *i*PS189 of *M.genitalium* [49]. A summary of the comparison is provided in Table 7.

**Table 7:** Comparison of the *Mycoplasma gentialium model (i*PS189) [49] and the model presented in this paper for *Mycoplasma gallisepticum*.

	*Mycoplasma genitalium* (*i*PS189)	*Mycoplasma gallisepticum*
Metabolites	276	362
Cytoplasmic	261	359
Extracellular	85	82
Reactions	349	395
Intracellular Rxn	178	234
Transport Rxn	84	86
Exchange Flux	87	73

The *M. gallisepticum* model was larger than *M. genitalium*, as was expected since genome size of *M.genitalium* is 0.58 Mb compare to 1.01 Mb of *M.gallisepticum*. The genome of *M. gallisepticum* had 817 genes that encoded for 763 proteins [74]; in contrast, *M.genitalium* had 524 genes that codified for 475 proteins [75]. The G+C content of *M.gallisepticum* is 31.5%, while the G+C content of *M. genitalium* is 32% [74].

The *M.genitalium* model had 46 fewer reactions than the *M.gallisepticum* model. However, it turns out that *i*PS189 model had more exchange fluxes. The additional exchange fluxes in the *M. genitalium* model were required to account for exchange of amino acids as a combination of dipeptides; *M.gallisepticum*, only had fluxes for amino acid exchange as monopeptides. The exchange of dipeptides in *M. gallisepticum* model was represented as a general reaction (trans-rxn7tv-3945) due to lack of information about specificity of this transporter.

Additionally, the *M.gallisepticum* model had 86 more metabolites than *i*PS189 model. These metabolites were mainly located in the cytosol space and included lipids. The *i*PS189 model did not have fully specified lipids in the biomass reaction or the associated lipid reactions. The *M. gallisepticum* model included cardiolipin, sphingomyelin, phosphatidylcholine, cholesterol, etc; and a set of reaction related with lipid metabolism such as phosphatidate phosphatase (E.C.3.1.3.40); phosphatidylglycerophosphatase (E.C.3.1.3.27); diacylglycerol chlorinephosphotransferase (E.C. 2.7.8.2); sphingomyelin synthase (E.C. 2.78.27); etc.

### *M. gallisepticum* model created with Model SEED

The RAST annotation server [76] was used to develop an annotated genome for SEED. Then Model SEED [77] was used to generate a preliminary reconstruction for *M. gallisepticum* model via the web-based resource (http://iris.kbase.us). During this process, Model SEED created a biomass reaction; the media chosen was a defined media already present in Model SEED. After, running the auto-completion tool of Model SEED, a growth rate of 2.02 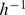 was predicted, the model had 358 reactions and 389 metabolites. When the *M. gallisepticum* model from SEED was constrained to the experimentally measured glucose uptake rate and lactate production rate, the model predicted no growth. It was not possible to use the model optimization tools of Model SEED as they were not available on the website at that time. As a result, a better fitting model incorporating the experimental growth data could not be created. Instead, GAFBA was used to perform a 2,000 generation run, resulting in a growth rate value of 2.5 h^−1^. However, the mass balances constraints of acetaldehyde, and dCMP had to be relaxed.

One more trial was done, using the *M.gallisepticum* model from Model SEED and adding the biomass reaction and media composition defined in this work without adding any of the lipid metabolites and tRNA charged/uncharged, since the *M.gallisepticum* model from Model SEED did not have them. The model generated had 354 reactions and 391 metabolites. The glucose uptake rate and lactate production rate were constrained to the experimental values, and again; no growth was predicted. GAFBA was used to evaluate the potential gaps of Model SEED. A 2,000 generation run was performed and resulting in a growth rate of 1.32 h^−1^, with 12 mass balance constraints being relaxed. These results indicate that GAFBA may be used with Model SEED to identify problematic metabolites.

### Frequency of relaxed constraints

The frequency of metabolites requiring relaxation of their mass balance constraint was determined for the best 20 models of the population. The rationale for such an analysis was that it might facilitate the identification of the most pathologic metabolites with respect to achieving a feasible solution to the optimization problem. The frequency analysis was done at simulation 3, 5, 17, 27, and the results are presented in Table 8. At simulation number three, 23 metabolites were shared among the pool of best 20 chromosomes; the best model only has one more metabolite dropped.

**Table 8:** Result of the analysis of relaxed mass balances constraints for select simulations.

Simulation number	Number of shared relaxed metabolic constraints in the best 20 chromosomes	Number of relaxed metabolic constraints in the best individual	Number of shared relaxed metabolic constraints already present in a previous simulation
3	23	24	NA
5	16	24	9
17	9	12	6
27	5	5	4

At simulation number five, 16 metabolites with relaxed mass balance constraints were common in the pool of best 20 chromosomes. By reviewing the list of metabolites, it was observed that nine metabolites were already dropped in simulation number 3, and four of them were charged/uncharged tRNAs. Thus, a recycling reaction for the charged/uncharged metabolites was added to the biomass equation.

Nine commons metabolites were found at simulation number 17 among the best 20 chromosomes. Six of them already appeared in either simulation number 3 or 5. One of them was ribose-5-phosphate, and by checking the metabolic pathway around this metabolite, the directionality of two reactions Ribulp3epim-Rxn (E.C. 5.1.3.1) and Rib5pisom-Rxn were changed to reversible (E.C. 5.3.1.6).

Finally, at simulation number 27, five metabolites were present in all the best 20 chromosomes, and four of them had previously appeared in the preceding simulations analyzed. At this point, the metabolites' studied were sodium ion and L-phosphatidate. The solution to the mass balance of sodium ion has already been discussed. The other metabolite L-phosphatidate was also part of the choline pathway. By adding the Na+ATPase reaction, the choline pathway was activated and the mass balance constraint for L-phosphatidate was closed.

## Discussion

The goal of this work was to develop an approach which could be used for the curation of metabolic networks and facilitate fundamental understanding and discovery of metabolism. To test this idea, a strategy was applied to the reconstruction of a genome-scale metabolic network for *M. gallisepticum*. By using GAFBA, it was possible to find gaps and inconsistencies present in the network that went beyond genome annotation. It was generally possible to fill these gaps based on the described heuristics and/or searching through the literature. Even when the process was not automated, time spent at the curation level was minimized because problematic metabolites were identified allowing one to focus only on the significant issues remaining.

An argument could be made for using optimization strategies other than a genetic algorithm, such as a mixed integer linear programming (MILP) approach, which MetaFlux, Model SEED, and GapFill use. Although the MILP approach is a powerful one, the GA method provides certain advantages such as scaling more effectively for the study of large networks, and generating a population of solutions that, as a whole, provide further insight into the problem domain.

A factor in favor of MILP is that MILP would potentially be faster than using GA. This is especially true for smaller models. However, even when the GA running time is greater than MILP, the computational time is not the rate-limiting step in the overall curation process. Rather the manual portion of the curation, such as the literature review and the assembly of the preliminary model, consume the bulk of the time.

In general, a GA based method may be seen as unnecessary for a small model such as the one developed in this work. However, it is anticipated that in the future, systems to be analyzed will grow significantly. Such growth may be due to organism complexity or due to dealing with microbial consortia. Under such circumstances, it is expected that GAFBA will be comparable or faster than MILP based methods since the computational complexity of GA falls within a range of O(n∧1.5) to O(n∧2), where *n* is the number of metabolites in the model [78]. In contrast, MILP computational complexity grows exponentially with system size [79], [80]. It should be further pointed out that GA's fall into the class of algorithms known as “embarrassingly parallel.” Although the approach presented does not currently take advantage of this feature, work is currently underway to parallelize the algorithm. With the advent of massively multi-core systems and especially GPU based computing, it is anticipated that significant gains in speed may be realized.

Another critical point and significant advantage regarding the GAFBA algorithm is the ability to carry out a population frequency analysis to better determine the most problematic metabolites. By observing the most frequently occurring relaxed constraints in that population, it was possible to identify candidate metabolites that were likely the most problematic. These results are extraordinarily valuable, as they provide the researcher with a starting point regarding which metabolites are most likely causing the model to fail.

Additionally, the GAFBA approach provides some important advantages over existing curation strategies. The most significant advantage is the possibility to identify problems without relying on a database for reference. For example, MetaFlux requires MetaCyc, Model SEED requires the SEED reaction database, and GapFill requires a customized multi-organism database. GAFBA provides flexibility by allowing identification and potential resolution of metabolic inconsistencies without requiring access to any type of database. It is important to realize, however, that GAFBA may be used in conjunction with the other approaches described, as was illustrated earlier with Model SEED. Thus the information from these databases is not lost. Rather, GAFBA may be used to complement existing approaches or it may be used on its own.

MetaFlux requests that the user set the values for the weights of the objective function in the MILP objective function. This step necessitates the proper understanding of the meaning of each weight. The GAFBA methodology does not need the user to define any weights. Thus, the set of metabolites to be studied will not depend on values defined by the user. In addition, MetaFlux does not have the ability to add reactions between compartments (e.g. transport reaction), resulting in additional level of complexity for curation purposes. GAFBA, however, does permit addition of reactions between compartments. Interestingly, MetaFlux does not give information about the metabolites that may be problematic; instead it provides a set of reactions to add in order to resolve metabolic inconsistencies. Conversely, GAFBA does not provide a list of reactions to add, but rather points out problematic metabolites that may be used to direct further research. These complementary approaches suggest that some synergy may be possible.

When comparing Model SEED with GAFBA, Model SEED provides a significant advantage in that it generates a biomass reaction and defined media for an organism. However, when the *M. gallisepticum* model created by Model SEED was used with the experimentally determined glucose and lactate constraints, the model predicted no growth, suggesting that more curation was needed. By applying GAFBA to the Model SEED generated model incorporating the experimental data, it was possible to identify the two problematic metabolic constraints inhibiting the viability of the model. This result serves to highlight GAFBA's role as a curation tool and its complementarity to existing model development software available.

In general, GAFBA provides an alternative and complementary method curation and modeling strategy to existing approaches. GAFBA could help to improve the quality of the existing metabolic networks and to generate new and more complete networks. Furthermore, because the approach used by GAFBA is complementary to existing approaches, it can be used after a model is created with Model SEED or MetaFlux.

With respect to *M. gallisepticum* specifically, the new genome-scale model generated using GAFBA replicated the experimentally observed data well. It is likely that the predicted growth rate was higher than the experimental value observed because the NGAM value and the GAM used in the model were based on the *M. genitalium i*PS189 model [49] and *M. pneumoniae* [51]. Further experiments are required to determine the appropriate NGAM value and GAM value for *M. gallispeticum*.

The results of GAFBA have highlighted a number of pursuable experimental avenues. For example, while GAFBA predicts the presence of formate dehydrogenase, NAD+ synthethase and Na-ATPase enzymes for *M. gallisepticum*, their presence still needs to be experimentally verified. The elucidation of MEP pathway role in *M. gallispecticum* metabolism may ultimately help to fundamental metabolic understanding of the organism. It is also important to determine if the MEP pathway is essential for the survival of *M. gallisepticum*, since it will help to elucidate possible drug targets as was recently illustrated in research for control of *Mycobacterium tuberculosis* [81], [82], *Haemophilus influenzae* [83], and in treatment against malaria [84], [85]

## Materials and Methods

### Genetic algorithm parameters

The genetic algorithm was implemented using an *elite selection* strategy [46]. The population consisted of 30 chromosomes. The crossover probability was set to 70%. The mutation probability was set to 1%. The GAFBA algorithm was run for 2,000 generations per simulation. The choice of 2,000 generations as the termination criteria was based on empirical observation. It was found that by 2,000 generations, the system generally reached a plateau, and no further changes in biomass and constraints were observed. Each simulation took around seven hours on a 3.33 GHz Intel Core 2 Duo CPU/4 GB workstation. Thus the total time for the 40 simulations was approximately 12 days.

### Flux balance analysis

The theory behind the development of FBA has been extensively discussed in the literature [86]–[93]; however, for the sake of completeness, a brief description is provided here. Mathematically, FBA is represented by the mass balance for each metabolite presented at the network under steady state conditions,
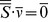
(1)

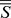
 represents the stoichiometric matrix, while 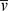 represents the flux.

To reduce the number of allowable solutions to this system of equations, a set of constraints is defined,
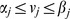
(2)

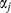
 and 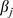 are the lower and upper bounds respectively of the 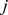 fluxes. The bounds for the reversible reactions are 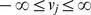, whereas for the irreversible reactions, they are 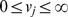. Since the media was undefined, the exchange fluxes had to be determined from literature information of relative species [49], [50], [94]. The boundaries for the exchange fluxes were defined for metabolites in the media as 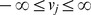, and for secretion of metabolites the following constraint was implemented: 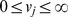 [49]. The lower boundary of the NGAM value was set to 8.4 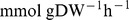 based on the value for the *M. genitalium i*PS189 model [49]. The upper and lower boundaries of the glucose uptake rate and lactate secretion rate were set to the experimentally determined values, which was −16.53 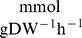 for glucose and 10.29 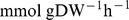 for lactate. The lower boundary value of the oxygen uptake rate was constrained to −43 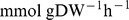
to avoid reaction-looping behavior [20], [34].

Finally, the optimal metabolic flux distribution was determined using the linear objective function,
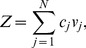
(3)where 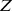 represents the objective function to be maximized, which in this case was the biomass equation for *M. gallisepticum*. The term 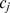 was a biologically determined coefficient that represented the contribution of 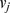 to the objective function 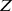.

### Strains, culture conditions

A previously sequenced clonal isolate of *M. gallisepticum* R_low_, R_low_ Clone 2 (RLC2) [74] was used. The bacteria were grown in complete Hayflick's medium [95] with an initial concentration 3.5 g/L glucose.

### Determining dry cell weight and CFU/ml

The CFU/ml concentrations for all experiments were calculated using a previously determined correlation [96] between CFUs/ml and the absorbance of cell culture at 620 nm. A correlation between dry cell weight and absorbance was generated by directly measuring the weight of dried cell pellet, volume of the supernatant, and OD_620_ of the culture. To accomplish this, 500 ml of RLC2 was grown in Hayflick's media [95] at 37°C to mid-log phase. The absorbance of the culture was measured at 620 nm. The culture was then quickly chilled to 4°C. 30 ml of the culture was placed in a 50 ml Falcon Tube and centrifuged at 15,000×g at 4°C for 15 mins. The supernatant was transferred to a graduated cylinder for measurement without disturbing the pellet, and another 30 ml of culture was added to the same tube and centrifuged at the conditions mentioned above. This was repeated until all 500 ml of culture had been centrifuged into one large pellet. The pellet in the 50 ml centrifuge tube was dried for two weeks at 37°C. The tube was then capped stored at 23°C for two months. The dried flaking pellet was then scraped off and measured using a scale accurate to the nearest mg. This protocol was repeated for four different cell concentrations in mid-log phase growth and one control using only medium.

### Metabolism experimental design

100 ml aliquots of Hayflick's media with 3.5 g/L of glucose in 250 ml plastic flat-bottom centrifuge tubes were inoculated with mid-log cultures of RLC2 and incubated at 37°C. Sampling of these vessels was initiated approximately 15–19 hours post-inoculation, when the dry cell density in each vessel reached 46.89 mg/L (7.27e7 CFU/ml) and ended once the dry cell density exceeded 125 mg/L. Samples of culture were taken at nearly one-hour intervals over the aforementioned period during late log-growth. This allowed for four to five individual sample points to be taken during this late log-phase growth for each run. From these samples, cell dry weight concentration, glucose concentration, and lactate concentration were determined by the assays described below. Seven runs were completed for RLC2. Three sets of runs were performed on separate days. Each set included approximately three RLC2 runs.

### Sampling and cell growth assay

10^6^ CFUs of RLC2 (growing in late log-phase) was added to 100 ml of media in a 250 ml plastic flat bottom centrifuge tube. This tube was incubated at 37°C while being shaken at 140 rpm for 15–19 hrs. 1 ml samples from each flat bottom centrifuge tube were then placed into 1.5 ml microfuge tubes. A 700 µL volume of each sample was immediately centrifuged at 20,000×g for 15 minutes at 4°C in an Eppendorf 5417 microfuge using a fixed-angle aerosol-tight rotor (FA-45-30-11) for 1.5–2.0 ml tubes.

The supernatant was transferred into new 1.5 ml microfuge tubes and placed in a −80°C freezer for storage until further analysis. The OD_620_ of the remaining 300 µL of each sample was measured while the other 700 µL was being centrifuged. This OD_620_ reading was used to determine the biomass concentration using the experimentally determined correlation between cell dry weight and OD_620_. This sampling process was repeated until the dry cell density in each vessel reached 125 mg/L. At this point, sampling ended and the remaining culture was allowed to grow overnight and was checked 24 hours later for visible signs of contamination. No contamination occurred.

### Glucose and lactate assays

After two to five days of storage at −80°C, the 700 µL supernatant samples were thawed and prepared for analysis using a YSI 2700 SELECT single-channel Biochemistry Analyzer. Supernatant samples were filtered with 13 mm GHP 0.2 micron syringe filters (WAT097962) to remove any remaining cellular debris. The supernatant samples were then placed in a −80°C freezer for another 1–7 days.

To assay for lactate, the standard YSI 2700 SELECT protocol for measuring L-lactate concentration using a L-lactate membrane (part # 2329) was followed. Standard buffer (part # 2357) and L-lactate calibration standard (part # 2776) were used. 25 µL of sample was taken and analyzed for a 30 second period before being flushed. Two readings were performed for each supernatant sample. Six supernatant samples at a time were thawed, assayed, and then refrozen to −80°C.

To assay for glucose, the standard YSI 2700 SELECT protocol for measuring glucose concentration using a glucose membrane (part # 2365) was followed. Standard buffer (part # 2357) and glucose calibration standard (part # 2776) were used. 25 µL of sample was taken and analyzed for a 30 second period before flush. For each supernatant sample, three replicates were performed and averaged. Six supernatant samples at a time were thawed, assayed, and then refrozen to −80°C.

### Dry weight measurement

The correlation between optical density and dry cell weight per ml is shown in Table S1. Consistent readings were achieved for four points within the range of optical densities covered by the metabolism experiment.

### Determination of biomass equation

The biomass equation was determined by incorporating all available published information about the makeup of *M. gallisepticum* and other similar mycoplasmas. The starting equation was taken from a metabolic reconstruction of *M. genitalium*, one of *M. gallisepticum*'s closest relatives [49]. Metabolites added in by the GrowMatch portion of that reconstruction were discarded due to lack of supporting evidence. Other cofactors not conducive with *Mycoplasma* metabolism that did not possess any cited synthesis or absorption pathways, such as Menaquinol 7, were modified or removed. The relative ratios of the various components of DNA, RNA, and amino acids reported in the *M. genitalium* reconstruction were kept due to the similar GC content and large number of shared proteins between the two organisms [97]. However, the ratios between the major biochemical components, consisting of DNA, RNA, amino acids, cofactors, ions, and lipids, were adjusted to reflecting a previously reported chemical composition analysis of *M. gallisepticum* [98].

The composition of the lipid fraction was calculated from published data on the lipid fractions of *M. gallisepticum* and other related *Mycoplasmas*. First, the ratios of the classes phospholipid, sterol, and triglyceride were determined relative to each other from previously published work [48], [98]. Though recent studies suggest that *M. gallisepticum* may possess a capsule, it has not been directly shown that its capsule or *M. gallisepticum* itself contains glycolipids [99]. Because of this uncertainty, and due to the small amounts of carbohydrates found in *M. gallisepticum*'s chemical composition relative to other related glycolipid possessing species, glycolipids were not included in the biomass composition [98].

The ratios of the subclasses of sterol and phospholipid groups relative to each other were then analyzed. First, sterols was analyzed. The amount of cholesterol and cholesterol ester was calculated using published ratios of cholesterol to cholesterol ester [48], [100].

Next, phospholipids were analyzed. The amount of sphingomyelin present in *M. gallisepticum*'s membrane was estimated from previously reported values [48], [100]. The percent of the phospholipid fraction made up of phosphatidylcholine was estimated by using a reported ratio between phosphatidylcholine and sphingomylin in *M. gallisepticum*'s membrane along with the reported sphingomyelin membrane fraction [48], [100]. The phosphatidic acid (1,2-diacylglycerol-3-phosphate) percent was estimated using a lipid percent reported by Tourtellotte *et al.* The amount of phosphatidic acid was decreased by 25% before incorporation into the biomass equation due to an author statement suggesting the value was higher than it should have been [48]. No conclusive assays of the existence or membrane percent of phosphatidylethanolamine have been published. Phosphatidyl-ethanolamine was shown to be absent by a complement fixing antigen assay of the membrane of *Mycoplasma pneumoniae* [101]. Because of the similarity between these two organisms, it was assumed that *M. gallisepticum* lacked this phospholipid as well. Tourtellotte *et al.* reported that phosphatidylglycerol likely made up less than 10% of the phospholipids. Since no definitive quantitative assay of phosphatidyl-glycerol have been performed on *M. gallisepticum* or any of its close relatives, the phospholipid percent of phosphatidyl-glycerol was estimated from a published value in *A. ladwaii* [102]. Cardiolipin's composition or existence in *M. gallisepticum* has yet to be studied. However, cardiolipin has been shown to be present in *Mycoplasma pneumoniae*'s membrane [103]. In a recent study, *M. pneumoniae* was shown to have the metabolic reactions necessary to biosynthesize cardiolipin from fatty acids and glycerol [51]. Cardiolipin is speculated to be the dominant phospholipid in other more distantly related *Mycoplasma* such as *M. mycoides* and *M. hyopneumonaie* [104]. Therefore, the remaining phospholipid fraction was assumed to be comprised primarily of cardiolipin. Supplementary information Table S2 summarizes the calculated composition of the lipid fraction used in the biomass equation.

The average molecular weight of *M. gallisepticum*'s phospholipids, sterols, and triglycerides R groups were by weighted averaging the molecular weights of the constituent fatty acids (supplementary information Table S3). The fatty acid compositions of each of the three lipid types were taken from a previously reported study [48]. Using these R group molecular weight estimations, the full lipid molecular weights were calculated using the chemical structures of the major lipid components (information Table S4). Using these molecular weights, the mmol/gDW values for each of the major lipid components were calculated from the mass percents shown in supplementary Table S2. The final biomass reaction is presented in the Data S1 supplementary file.

## Supporting Information

Data S1 **A description of the FBA model for** ***M. gallisepticum*** **in Excel format.**
(XLS)

Data S2 **A description of the FBA model for** ***M. gallisepticum*** **in SBML format.**
(TXT)

Data S3 **The source code of the** ***M. gallisepticum*** **model for use with GLPK.**
(TXT)

Figure S1 **Case 1. Change directionality of reactions.** The purple circle showed the dropped metabolite. The blue arrows are the fluxes and the orange arrows are the proposed solutions. The solid blue line is the plasma membrane and the dashed blue line is the system boundary.
(TIFF)

Figure S2 **Case 2. Add exchange flux.** The purple circle showed the dropped metabolite. The blue arrows are the intracellular fluxes and the orange arrow is the proposed solutions. The solid blue line is the plasma membrane and the dashed blue line is the system boundary. The red circle represents the Biomass pool.
(TIFF)

Figure S3 **Case 3. Add reaction.** (formate case). The purple circle showed the dropped metabolite. The blue arrows are the fluxes, the blue thick arrows are the exchange fluxes, and the orange arrow is the proposed solutions. The solid blue line is the plasma membrane and the dashed blue line is the system boundary. The red circle represents the Biomass pool.
(TIFF)

Table S1 **OD/(g/cfu) correlation.** Calculations of the g/cfu conversion.
(DOCX)

Table S2 **Lipid fraction estimation.** Percent composition of the lipid fraction of *M. gallisepticum*, the estimated molecular weights, and the relevant references used to generate these percentages.
(DOCX)

Table S3 **Average fatty acid molecular weight.** The calculation of the average fatty acid molecular weight from the fatty acid composition of *Mycoplasma gallisepticum* as reported by Tourtelloute et al. [48].
(DOCX)

Table S4 **Estimation of molecular weights for major lipid components making up biomass.** Using the chemical structure, the molecular weights of each molecule, and the estimated molecular weights of the R group, whose calculations are shown in Table S3, the molecular weight of each major lipid component was estimated.
(DOCX)

Text S1 **Examples of methodologies for resolving infeasible models.**
(DOCX)
