# Supplementary figures and images for: Semi-automated Curation of Metabolic Models via Flux Balance Analysis: A Case Study with Mycoplasma gallisepticum
Source: PLoS Comput Biol. 2013 Sep 5;9(9):e1003208. doi: 10.1371/journal.pcbi.1003208 (PMC3764002; doi:10.1371/journal.pcbi.1003208)

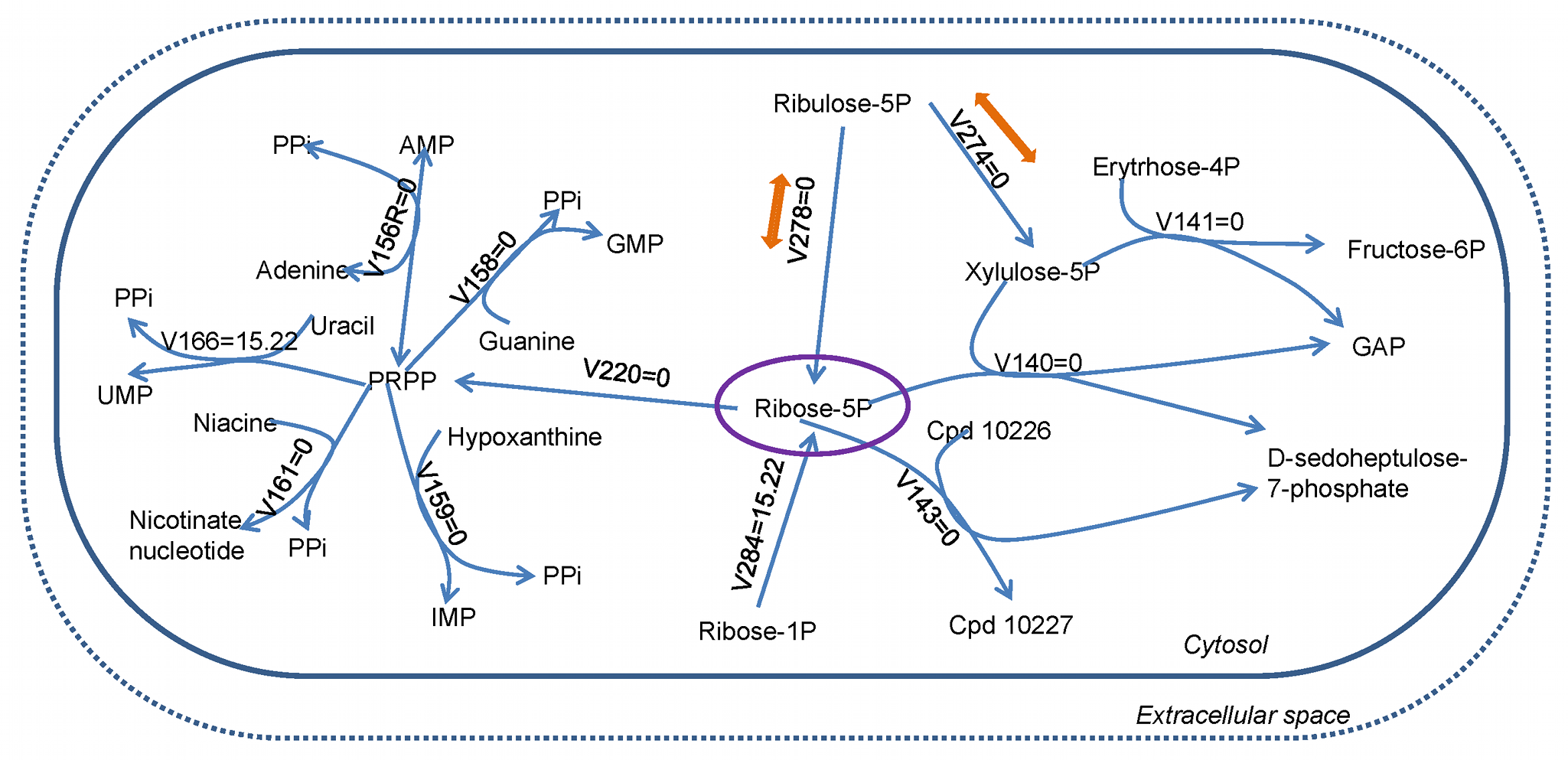

Supplement: Figure S1 — Case 1. Change directionality of reactions. The purple circle showed the dropped metabolite. The blue arrows are the fluxes and the orange arrows are the proposed solutions. The solid blue line is the plasma membrane and the dashed blue line is the system boundary. (TIFF) [file pcbi.1003208.s004.tif]

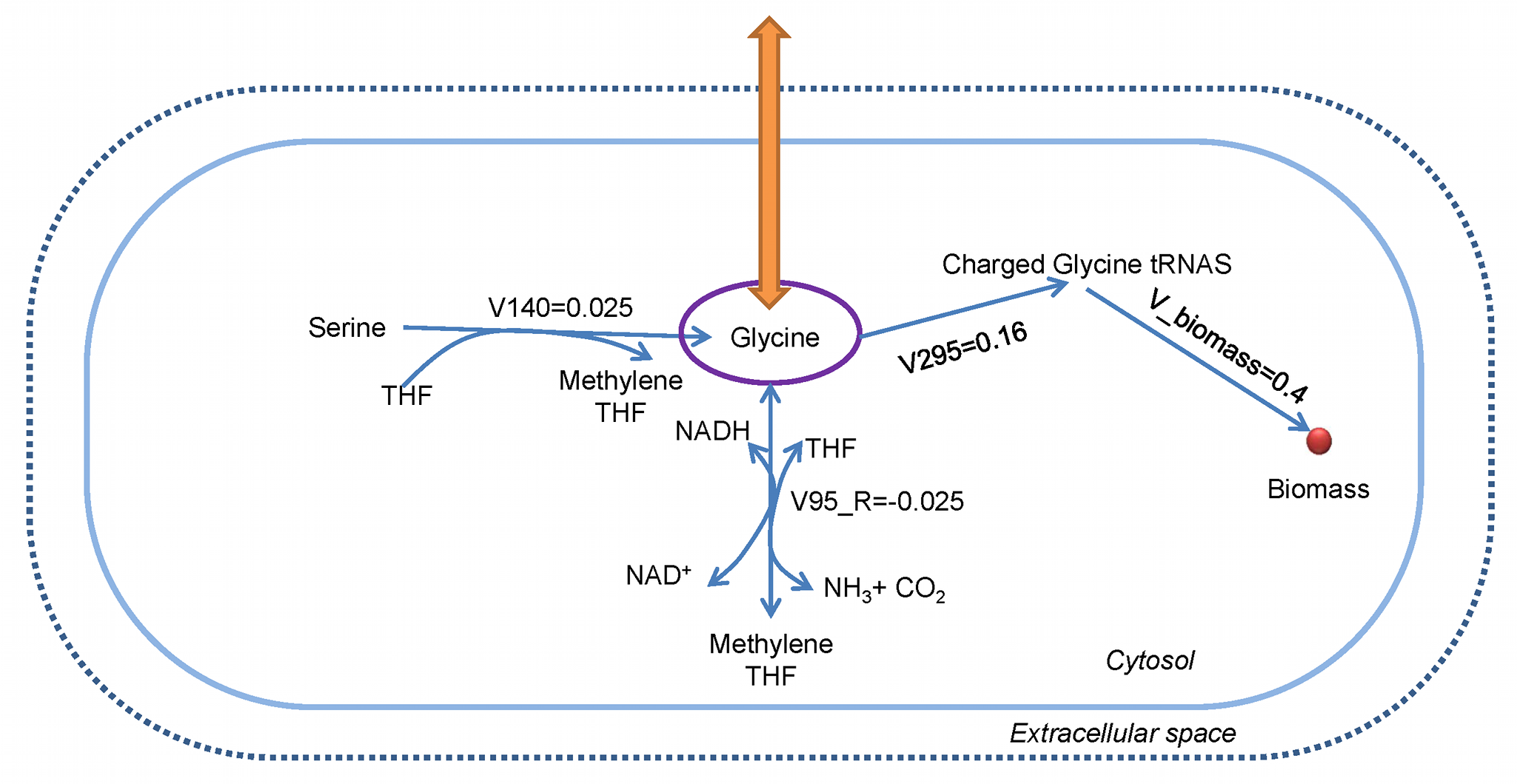

Supplement: Figure S2 — Case 2. Add exchange flux. The purple circle showed the dropped metabolite. The blue arrows are the intracellular fluxes and the orange arrow is the proposed solutions. The solid blue line is the plasma membrane and the dashed blue line is the system boundary. The red circle represents the Biomass pool. (TIFF) [file pcbi.1003208.s005.tif]

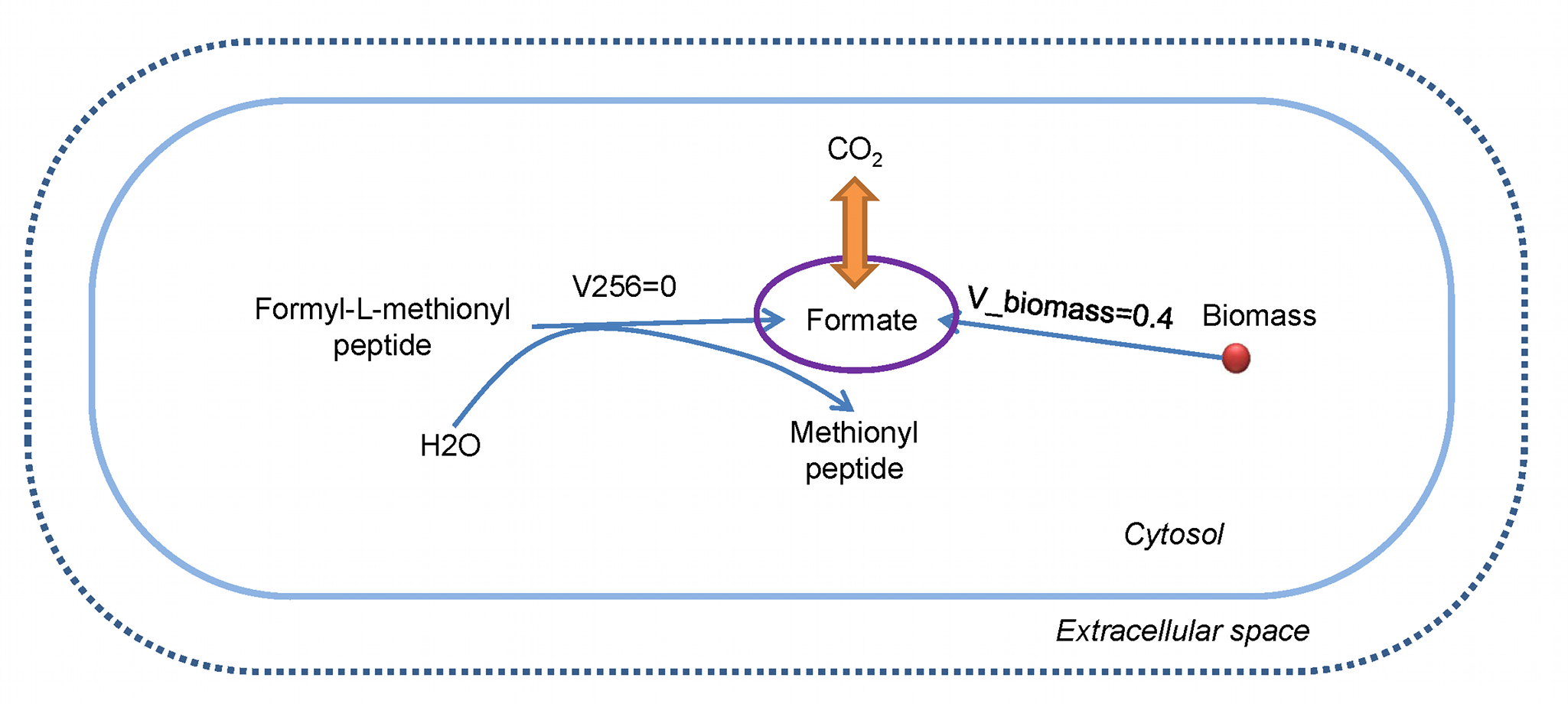

Supplement: Figure S3 — Case 3. Add reaction. (formate case). The purple circle showed the dropped metabolite. The blue arrows are the fluxes, the blue thick arrows are the exchange fluxes, and the orange arrow is the proposed solutions. The solid blue line is the plasma membrane and the dashed blue line is the system boundary. The red circle represents the Biomass pool. (TIFF) [file pcbi.1003208.s006.tif]
