# Supplementary material for: Semi-automated Curation of Metabolic Models via Flux Balance Analysis: A Case Study with Mycoplasma gallisepticum
Source: PLoS Comput Biol. 2013 Sep 5;9(9):e1003208. doi: 10.1371/journal.pcbi.1003208 (PMC3764002; doi:10.1371/journal.pcbi.1003208)
Supplement: Table S1 — OD/(g/cfu) correlation. Calculations of the g/cfu conversion. (DOCX) [file pcbi.1003208.s007.docx]

**Table S1. OD/(g/cfu) Correlation.** Calculations of the g/cfu conversion.

| Run | OD_620_ | Pellet Weight (g) | Supernatant Volume (ml) | Concentration (g/ml) | cfu/ml | g/cfu |
| --- | --- | --- | --- | --- | --- | --- |
| Control | 0 | 0 | 432 | 0.000E+00 | N/A | N/A |
| 1 | 0.089 | 0.051 | 478 | 1.067E-04 | 1.66E+08 | 6.44E-13 |
| 2 | 0.084 | 0.05 | 480 | 1.042E-04 | 1.56E+08 | 6.68E-13 |
| 3 | 0.098 | 0.053 | 480 | 1.146E-04 | 1.83E+08 | 6.26E-13 |
| 4 | 0.094 | 0.052 | 482 | 1.120E-04 | 1.75E+08 | 6.39E-13 |
|  |  |  |  |  | average: | 6.45E-13 |
|  |  |  |  |  | std: | 1.75E-14 |
