# Supplementary material for: Semi-automated Curation of Metabolic Models via Flux Balance Analysis: A Case Study with Mycoplasma gallisepticum
Source: PLoS Comput Biol. 2013 Sep 5;9(9):e1003208. doi: 10.1371/journal.pcbi.1003208 (PMC3764002; doi:10.1371/journal.pcbi.1003208)
Supplement: Table S2 — Lipid fraction estimation. Percent composition of the lipid fraction of M. gallisepticum, the estimated molecular weights, and the relevant references used to generate these percentages. (DOCX) [file pcbi.1003208.s008.docx]

**Table S2. Lipid Fraction Estimation.** Percent composition of the lipid fraction of *M. gallisepticum*, the estimated molecular weights, and the relevant references used to generate these percentages.

| Lipid Ttype | %of Total Lipid Mass | Lipid Name | MW | Mass % of Lipid Type | g/gDW | Citations |
| --- | --- | --- | --- | --- | --- | --- |
| phospholipid | 70.3 |  |  |  |  | [[1](#_ENREF_1)] |
|  |  | cardiolipin | 1524.7 | 49.5 | 0.0546 | [[2-5](#_ENREF_2)] |
|  |  | 1,2-diacylglycerol-3-phosphate | 651.3 | 7.5 | 0.0083 | [[1](#_ENREF_1)] |
|  |  | phosphatidyl-choline | 819.5 | 23.8 | 0.0283 | [[1](#_ENREF_1),[5](#_ENREF_5)] |
|  |  | sphingo-myelin | 771.8 | 9.2 | 0.0102 | [[1](#_ENREF_1)] |
|  |  | phosphatidyl-ethanolamine | 776.4 | 0 | 0.0000 | [[1](#_ENREF_1),[6](#_ENREF_6)] |
|  |  | phosphatidyl-glycerol | 807.4 | 10 | 0.0110 | [[1](#_ENREF_1),[7](#_ENREF_7)] |
| Sterols | 21.9 |  |  |  |  | [[1](#_ENREF_1),[8](#_ENREF_8)] |
|  |  | cholesterol | 386.7 | 90.6 | 0.0312 | [[5](#_ENREF_5),[9](#_ENREF_9)] |
|  |  | cholesterol ester | 643.7 | 9.3 | 0.0032 | [[9](#_ENREF_9)] |
| triglycerides | 7.7 |  |  |  |  | [[1](#_ENREF_1)] |
|  |  | triacylglyceride | 794.1 | 100 | 0.0121 | [[1](#_ENREF_1),[5](#_ENREF_5)] |
| glycolipids | 0 |  |  |  |  | [[10](#_ENREF_10)] |

**Reference**

1. Tourtellotte ME, Jensen RG, Gander GW, Morowitz HJ (1963) Lipid Composition and Synthesis in the Pleuropneumonia-Like Organism *Mycoplasma Gallisepticum*. J Bacteriol 86: 370-379.

2. Yus E, Maier T, Michalodimitrakis K, van Noort V, Yamada T, et al. (2009) Impact of genome reduction on bacterial metabolism and its regulation. Science 326: 1263-1268.

3. Snowden N, Wilson PB, Longson M, Pumphrey RS (1990) Antiphospholipid antibodies and *Mycoplasma pneumoniae* infection. Postgrad Med J 66: 356-362.

4. Arraes FBM, Carvalho MJAd, Maranhão AQ, Brígido MM, Pedrosa FO, et al. (2007) Differential metabolism of *Mycoplasma* species as revealed by their genomes. Genetics and Molecular Biology 30: 182-189.

5. Rottem S, Markowitz O (1979) Membrane lipids of *Mycoplasma gallisepticum*: a disaturated phosphatidylcholine and a phosphatidylglycerol with an unusual positional distribution of fatty acids. Biochemistry 18: 2930-2935.

6. Beckman BL, Kenny GE (1968) Immunochemical analysis of serologically active lipids of *Mycoplasma pneumoniae*. J Bacteriol 96: 1171-1180.

7. Gross Z, Rottem S (1979) Lipid distribution in *Acholeplasma laidlawii* membrane. A study using the lactoperoxidase-mediated iodination. Biochim Biophys Acta 555: 547-552.

8. Razin S, Argaman M, Avigan J (1963) Chemical Composition of *Mycoplasma* Cells and Membranes. J Gen Microbiol 33: 477-487.

9. Argaman M, Razin S (1965) Cholesterol and Cholesterol Esters in Mycoplasma. J Gen Microbiol 38: 153-160.

10. Langworthy TA (1983) Lipid tracers of mycoplasma phylogeny. Yale J Biol Med 56: 385-390.
