# Supplementary material for: Semi-automated Curation of Metabolic Models via Flux Balance Analysis: A Case Study with Mycoplasma gallisepticum
Source: PLoS Comput Biol. 2013 Sep 5;9(9):e1003208. doi: 10.1371/journal.pcbi.1003208 (PMC3764002; doi:10.1371/journal.pcbi.1003208)
Supplement: Table S3 — Average fatty acid molecular weight. The calculation of the average fatty acid molecular weight from the fatty acid composition of Mycoplasma gallisepticum as reported by Tourtelloute et al. [48]. (DOCX) [file pcbi.1003208.s009.docx]

**Table S3. Average Fatty Acid Molecular Weight.** The calculation of the average fatty acid molecular weight from the fatty acid composition of *Mycoplasma gallisepticum* as reported by Tourtelloute et al. [[1](#_ENREF_1)]

|  | MW (g/mol) | Phospholipids % | cholesterol esters % | triglycerides % |
| --- | --- | --- | --- | --- |
| Caproic | 116.16 | 0 | 0 | 0.6 |
| Caprylic | 144.22 | 0 | 0 | 0.9 |
| Capric | 172.27 | 0 | 0 | 6.1 |
| Unidentified | 172.27 | 0 | 0 | 0.6 |
| Lauric | 200.32 | 0.7 | 0 | 6.3 |
| Myristic | 228.36 | 3.3 | 1.2 | 14.3 |
| Myrisroleic | 240.4 | 2.2 | 0.3 | 1.4 |
| Pentadecanoic | 242.4 | 0 | 0.3 | 1.1 |
| Palmitic | 256.43 | 24.8 | 23.4 | 32.2 |
| Palmitoleic | 256.43 | 2.9 | 6.3 | 1.1 |
| Heptadecanoic | 270.45 | 2.4 | 1.2 | 0.5 |
| Stearic | 284.48 | 36 | 13.1 | 10.1 |
| Oleic | 282.47 | 20.4 | 54.2 | 21.9 |
| Linoleic | 278.43 | 7.3 | 0 | 3.9 |
|  |  |  |  |  |
| Average Fatty Acid MW |  | 272.1 | 274 | 251 |

**Reference**

1. Tourtellotte ME, Jensen RG, Gander GW, Morowitz HJ (1963) Lipid Composition and Synthesis in the Pleuropneumonia-Like Organism *Mycoplasma Gallisepticum*. J Bacteriol 86: 370-379.
