# Supplementary material for: Semi-automated Curation of Metabolic Models via Flux Balance Analysis: A Case Study with Mycoplasma gallisepticum
Source: PLoS Comput Biol. 2013 Sep 5;9(9):e1003208. doi: 10.1371/journal.pcbi.1003208 (PMC3764002; doi:10.1371/journal.pcbi.1003208)
Supplement: Table S4 — Estimation of molecular weights for major lipid components making up biomass. Using the chemical structure, the molecular weights of each molecule, and the estimated molecular weights of the R group, whose calculations are shown in Table S3, the molecular weight of each major lipid component was estimated. (DOCX) [file pcbi.1003208.s010.docx]

**Table S4. Estimation of molecular weights for major lipid components making up biomass.** Using the chemical structure, the molecular weights of each molecule, and the estimated molecular weights of the R group, whose calculations are shown in Table S3, the molecular weight of each major lipid component was estimated.

| Component | MW | a 1,2-diacylglycerol-3-phosphate | a phosphotidyl-choline | cardiolipin |  |
| --- | --- | --- | --- | --- | --- |
| O | 16 | 8 | 8 | 17 |  |
| C | 12.01 | 5 | 10 | 13 |  |
| H | 1.01 | 7 | 19 | 20 |  |
| P | 82 | 1 | 1 | 2 |  |
| N | 14 | 0 | 1 | 0 |  |
| R groups | 228.1 | 2 | 2 | 4 |  |
| Estimated Total MW |  | 733.32 | 819.49 | 1524.73 |  |
| Component | MW | a sphingomyelin | an L-1-Phosphatidyl-ethanolamine | an L-1-phosphatidylglycerol |  |
| O | 16 | 6 | 8 | 10 |  |
| C | 12.01 | 24 | 7 | 8 |  |
| H | 1.01 | 49 | 12 | 13 |  |
| P | 82 | 1 | 1 | 1 |  |
| N | 14 | 2 | 1 | 0 |  |
| R groups | 228.1 | 1 | 2 | 2 |  |
| Estimated Total MW |  | 771.83 | 776.39 | 807.41 |  |
| Component | MW | cholesterol | a cholesterol ester | MW | a triacylglycerol |
| O | 16 | 1 | 2 | 169 | 6 |
| C | 12.01 | 27 | 28 | 12.01 | 6 |
| H | 1.01 | 46 | 45 | 1.01 | 5 |
| P | 82 | 0 | 0 | 82 | 0 |
| N | 14 | 0 | 0 | 14 | 0 |
| R groups | 230 | 0 | 1 | 207 | 3 |
| Estimated Total MW |  | 386.73 | 643.73 |  | 794.11 |
