# Supplementary material for: Semi-automated Curation of Metabolic Models via Flux Balance Analysis: A Case Study with Mycoplasma gallisepticum
Source: PLoS Comput Biol. 2013 Sep 5;9(9):e1003208. doi: 10.1371/journal.pcbi.1003208 (PMC3764002; doi:10.1371/journal.pcbi.1003208)
Supplement: Text S1 — Examples of methodologies for resolving infeasible models. (DOCX) [file pcbi.1003208.s011.docx]

**Supplementary information**

**Curation Algorithm**

Generally, four types of solution for the unbalanced metabolites can resolve curation issues.

Case 1: The change of the directionality of a reaction may solve the problem. Figure S1 has an example of this case. The ribulose-5 phosphate did not have a reaction to be produced and the mass balance of ribose- 5 phosphate was dropped, because any of the possible reactions to consume it were inactivated. If the reaction v278 catalyzed by the enzyme ribose-5-phosphate isomerase (E.C. 5.3.1.6) was changed to reversible, it could consume the ribose-5-phosphate.

From the Kyoto Encyclopedia of Genes and Genomes (KEGG) (Kanehisa et al., 2006), this reaction does indeed appear to be reversible, along with reaction v274 catalyzed by the enzyme ribulose-phosphate 3-epimerase (E.C. 5.1.3.1).

Case 2. The addition of an exchange flux may resolve the issue. In the case highlighted in Figure S2, glycine was produced by two reactions involved in folate transformations: V140 (GLYOHMETRANS-RXN E.C. 2.1.2.1) and V_95R (GCVMULTI-RXN in Pathway tools nomenclature). However, these reactions were unable to produce all the glycine required for the tRNA charging reaction, thus the additional glycine had to be taken from the media. The literature reported the presence of glycine in the media of the *Mycoplasma laidlawii* and the *Mycoplasma genitalium*, related species to the *M. gallispeticum*  (Suthers, et al., 2009; Tourtellotte, et al., 1964).

Case 3: Figure S3 shows an example for the case when the addition of a reaction could help to complete the mass balance of the metabolite. Here, the formate was produced via reaction V256. The peptide deformylase enzyme (E.C. 3.5.1.88) catalyzed this reaction and the biomass reaction. However, no reaction consuming formate was present. Therefore, a simple degradation pathway present in other Mycoplasmas (Yus et al, 2009) was added to the model.

Case 4: Finally, removing a reaction or metabolite from model may resolve the issue. On some occasions, the deletion of a general reaction or general metabolite from the model was required to fulfill the mass balance. These general reactions and metabolites were present in the model based on some of the software tools used to generate the initial model from the genome annotation. Usually, a general reaction is first created, and as the experimental data and information are available to confirm the presence of the specific reactions, the general reaction is expanded to the specific ones. Occasionally this may require the manual intervention of the curator.
